# Genomic Regions and Floral Traits Contributing to Low Temperature Tolerance at Young Microspore Stage in a Rice (*Oryza sativa* L.) Recombinant Inbred Line Population of Sherpa/IRAT109

**DOI:** 10.3389/fpls.2022.873677

**Published:** 2022-04-29

**Authors:** Ricky Vinarao, Christopher Proud, Peter Snell, Shu Fukai, Jaquie Mitchell

**Affiliations:** ^1^School of Agriculture and Food Sciences, The University of Queensland, Brisbane, QLD, Australia; ^2^Department of Primary Industries, Yanco Agricultural Institute, Yanco, NSW, Australia

**Keywords:** aerobic rice, floral traits, low temperature stress, QTL, *OsMADS50*, whole genome re-sequencing

## Abstract

Aerobic rice production (AP) consumes less water compared to flooded systems. Developing genotypes and identifying genomic regions associated with low temperature (LT) tolerance at the young microspore stage (YMS) is imperative for AP, particularly for temperate regions. Using a recombinant inbred line population derived from the Australian LT tolerant variety Sherpa, experiments were conducted to map and dissect quantitative trait loci (QTL) associated with spikelet sterility (SS) after exposure to LT and to investigate floral traits contributing to the development of lower SS. Significant genotypic variation for SS was observed in the population after exposure to LT at YMS. Three genomic regions associated with SS, *qYMCT3*, *qYMCT4*, and *qYMCT8.1* were identified in chromosomes 3, 4, and 8 respectively, using multiple QTL models explaining 22.4% of the genotypic variation. Introgression of the favorable allele from *qYMCT3* was estimated to reduce SS by up to 15.4%. A co-locating genomic region with *qYMCT3*, *qDTHW3.1* was identified as the major QTL affecting days to heading and explained as much as 44.7% of the genotypic variation. Whole-genome sequence and bioinformatic analyses demonstrated *OsMADS50* as the candidate gene for *qYMCT3/qDTHW3.1* and to our knowledge, this was the first attempt in connecting the role of *OsMADS50* in both LT and flowering in rice. Differential sets selected for extreme SS showed LT tolerant genotype group produced higher total pollen per spikelet resulting in a higher number of dehisced anthers and pollen on stigma and eventually, lower SS than THE sensitive group. The relationship between these key floral traits with SS was induced only after exposure to LT and was not observed in warm ideal temperature conditions. Identification of elite germplasm with favorable QTL allele and combinations, gene cloning, and pyramiding with additional high-value QTL for key traits should empower breeders to develop AP adapted genotypes for temperate growing regions, and ultimately produce climate-resilient rice.

## Introduction

Rice (*Oryza sativa* L.) is the world’s most important food crop and a primary food source for more than half of the world’s population (Khush, 2005). It is grown worldwide under a range of agro-climatic conditions in both tropical and temperate regions. Exposure of rice to low temperature (LT) affects the crop at all growth stages from germination to maturity. LT impairs seed germination, reduces seedling vigor, reduces plant height, delays heading, increases the production of degenerated spikelets, and decreases spikelet fertility. In temperate regions, LT stress at the reproductive stage, particularly in the young microspore stage (YMS) (Satake and Hayase, 1970; Susanti et al., 2019) is a major limiting factor the for adaptation of cultivars (Xu et al., 2008). Exposure of rice to LT (≤15°C) at the YMS increases spikelet sterility directly affecting grain yield, and therefore, LT tolerance at YMS is one of the most important targets for varietal improvement and development programs. LT during the reproductive stage of rice has significantly reduced rice productions in many temperate countries including Korea, China, Japan, and Australia (Lee, 2000; Farrell et al., 2001; Xu et al., 2008; Ahamed et al., 2012). In Korea, a maximum yield loss of milled rice of 3.9 t/ha was recorded during 1980 and has damaged 20% of the total rice area in 1993. Similarly, China accounts 3–5 million tons of production loss due to LT stress annually (Zhu et al., 2016). Japan also recorded rice crop damage equivalent to 158 billion yen in 2009 due to LT stress (Ahamed et al., 2012). In Australia, rice production is concentrated in the southern parts of the country- in the New South Wales’ Riverina irrigation areas. Rice in southern Australia is subjected to low humidity and large variations in diurnal temperature (Farrell et al., 2006a). An annual yield reduction of 0.68 t/ha was estimated for the Australian rice industry due to LT stress and was estimated to cost $20 million a year (Farrell et al., 2001). Yield loss of 1–2 t/ha was recorded in Australia during 1995–1996 due to low temperature at the reproductive stage, and a yield decline of more than 2 t/ha was recorded during the 2003–2004 cropping season (ABARES, 2021).

The aerobic production (AP) system is a relatively new water-saving echnology and is an intensive rice cultivation method that involves direct seeded rice cultivation under non-flooded, well-watered conditions (Kato et al., 2009). To successfully produce aerobic varieties for southern Australian environments and other temperate rice growing regions, genotypes adapted to the specific local conditions should be identified. Currently, rice growers ensure that permanent water (PW) with a depth of ∼25–30 cm is achieved and maintained after panicle initiation, to act as a blanket/buffer for the temperature fluctuations. As this buffer is removed in AP systems, genotypes are exposed to the detrimental effects of LT conditions. Low temperature is thus a limiting factor for the adoption of AP in Australia, particularly as genotypes grown in AP systems show higher levels of LT sensitivity, compared to PW systems (Ha et al., 2018). Consequently, genotypes must be developed for AP systems in temperate environments that possess a higher level of LT tolerance than typically required in PW systems. The development of these genotypes will not only protect the crop from damage brought about by low temperatures but will also support the water-saving ability of AP systems.

Several genetic studies have been conducted to map genomic regions associated with LT at the reproductive stage. Quantitative trait loci (QTL) linkage analysis has been conducted using various mapping populations such as near-isogenic lines (NILs), recombinant inbred lines (RILs), and doubled haploid (DH), and F_2_. *Ctb1* and *Ctb2* were mapped from chromosome 4 using NIL derived from Norin PL8 as the donor. It was subsequently fine-mapped into a 17-kb region and Saito et al. (2010) suggested that the *Ctb1* gene functions as a part of the E3 ubiquitin ligase complex. Xu et al. (2008) used Kunmingxiaobaigu, a japonica landrace, as a donor to identify eight QTL across chromosomes 4, 5, 10, and 11 using a BC_5_F_2_ population. Utilizing RILs derived from M202, Andaya and Mackill (2003) identified QTL *qCTB2a* and *qCTB3* on chromosomes 2 and 3, respectively. More recently, three main effects QTL (*qPSST-3*, *qPSST-7*, and *qPSST-9*) were also mapped and identified using a RIL population derived from IR66160-121-4-4-2 (LT tolerant donor) and Geumobyeo (LT-sensitive cultivar) genotyped using simple sequence repeat markers (Suh et al., 2010). Takeuchi et al. (2001) used a DH population from a cross between Akihikari (LT-sensitive) and Koshihikari (LT-tolerant) and genotyped using restriction fragment length polymorphism and randomly amplified polymorphic DNA markers. Three QTL for LT tolerance were identified, *qCT-1*, *qCT-7*, and *qCT-11*, and were located on chromosomes 1, 7, and 11, respectively (Takeuchi et al., 2001).

Low-temperature stress not only causes physical and visible damage to rice plants but also causes changes at the physiological level of the crop. Physiological fluctuations include electrolyte leakage, changes in the chlorophyll fluorescence, an increase in the relative amounts of stress-related metabolites, and the production of compatible solutes (Zhang et al., 2014). Reactive oxygen species (ROS) are produced in normal plant cellular metabolism and more so when crops are exposed to biotic and abiotic stress. A possible link between these physiological parameters may be that compromised ROS scavenging activity may lead to membrane lipid peroxidation causing extreme changes in cellular oxidative balance. Microspore development is in turn affected by this shift in oxidative balance (de los Reyes et al., 2018) causing irreversible damage to the formation of pollen grains, and may provide a link between these biochemical changes and key floral traits for LT tolerance at YMS. Sherpa an Australian variety that was released in 2011 (Snell and Reinke, 2011), can tolerate temperatures two degrees Celsius lower than previously existing varieties,^1^ and continues to be the LT tolerant check variety for the industry. To date, limited physiological and genetic studies have been conducted to investigate Sherpa’s LT tolerance mechanisms and related genomic regions. The aim of this study was to (1) map and dissect genomic regions associated with LT tolerance using Sherpa as the donor and (2) characterize floral traits and mechanisms for reproductive stage LT tolerance from Sherpa.

## Materials and Methods

### Plant Materials and Growing Conditions

Progeny (262 RILs) between Sherpa and IRAT109 were produced (F_1_s on two separate occasions) by the NSW Department of Primary Industries (DPI). The resulting F_1_s were then self-pollinated to produce F_2_s and subsequently, single seed descent method was carried out until F_6_ generation to produce the RILs. Black plastic tube pots were filled with Lockyer prairie soil consisting of light, black clay [USDA Soil Taxonomy: Fluventic Hapludolls; (Isbell, 2016)] across all the experiments carried out in this study. Fertilizers were added at a rate of 3 kg/m^3^ of slow release Osmocote^®^ 3–4M (19-9-10 + 2MgO + TE, ICL Specialty Fertilizers, Australia) and 2 kg/m^3^ of slow release Osmocote^®^ 5–6M (15-9-12 + 2MgO + TE) which translated to 0.72 and 0.48 g of each fertilizer per pot (0.24 L), respectively. Each pot was encased with a similar sized pot to prevent root escape. Approximately five seeds were sown in each pot and were subsequently thinned to a density of one seedling per pot 18 days after seeding (DAS). Seedlings were grown in an aerobic condition until 11 DAS, after which pots were gradually flooded with water level raised by 3 cm until the seedlings were fully flooded (3 cm above the soil surface). Iron sulfate and insecticide were sprayed as needed.

### Treatment and Phenotypic Characterization of the Full Population

Alongside the 262 RILs, 26 checks were added in the experiments including known LT tolerant (Lijiangheigu and RL11) and susceptible (Reiziq and RL206) checks and other varieties and advanced breeding lines. The LT screening protocol was carried out as outlined by Mitchell et al. (2016) with some modifications. The 288 lines were seeded in two sets: Set 1 (Experiment 1) and Set 2 (Experiment 2). Experiment 1 was a partially replicated design. Experiment 2 was planted 16 days after Experiment 1 and was designed with three full replications. Both experiments were grown in a warm room (30°C/21°C day/night) with natural light. When a single plant of a particular genotype reached heading (when the first spikelet emerged from the sheath) in Experiment 1, the corresponding plants in Experiment 2 were moved to a low temperature room (20°C/15°C day/night) for 14 days for LT treatment. The plants were then moved back to the warm room until maturity. At maturity, plants in Experiment 2 were phenotyped for the following traits: plant height (PH), spikelet per panicle (SPP), and spikelet sterility (SS). Total number of spikelets were counted from mainstem panicles, for which filled (FS), unfilled, and dead spikelets were identified and counted independently. SS was determined using the ratio between the sum of unfilled and dead spikelets over the total number of spikelets multiplied by 100. Days to heading (DTH) for Experiment 1 genotypes were also recorded and analyzed.

The full population was re-evaluated for LT tolerance similar to the above experiments conducted with some modifications to confirm consistency of identified genomic regions associated with LT tolerance traits. Set 1 (Experiment 3) and Set 2 (Experiment 4) were seeded 16 days apart with two replications in each experiment in a temperature and light controlled glasshouse. Both sets were grown in a warm room (28°C/22°C day/night) and light was controlled with installation of black-out curtain from 5:00 PM to 7:00 AM. Corresponding plants from Experiment 4 were moved to a low temperature room (21°C/15°C day/night) when a single replicate from Experiment 3 reached heading and subsequently, the same steps were carried out as described in Experiments 1 and 2. DTH and SS from Experiments 3 and 4, respectively were collected and used for QTL mapping.

### Treatment and Phenotypic Characterization of the Differential Set

From the results of Experiments 1 and 2, a set of differential RILs which represented the phenotypic extremes in terms of SS of the population were selected to determine the responses of SS and floral traits to LT in Experiment 5 and 6. The RIL selection was based on DTH from the full population evaluation (<70, 70–75, 75–80, and >80 days after seeding), with tolerant and sensitive RILs having SS < 32% and SS > 68%, respectively. For each of these maturity groups, the top five and bottom five genotypes in terms of SS were selected. A further nine genotypes were added based on their relatively low SS from Experiment 2 (<32%) and along with two promising RILs (with other useful AP traits) bringing the total number of RILs to 51. Additionally, nine check genotypes (parents Sherpa and IRAT109, known LT tolerant donors – Norin PL8, Lijiangheigu, and RL11, and elite genotypes – Reiziq, YRE16 = V71, Viand, and YUA15 = V037) were also included. Using the two-set system described above, these 60 genotypes each with three replications were grown in warm conditions throughout the growing period (30°C/21°C day/night) in Experiment 5, while in Experiment 6 they were exposed to low temperature conditions (20°C/15°C day/night) at YMS for 14 days.

Spikelet sterility, DTH, and PH of the genotypes from Experiments 5 and 6 were recorded. Main stems in all the plants tested were tagged and subsequent collection of spikelets for floral trait analyses were obtained from the panicle of the same stem. Floral traits including number of pollen on stigma (POS), number of dehisced anthers (NDA), and total number of pollen per spikelet (TP) using an impedance flow cytometer (Amphasys AG, Switzerland) were quantified. For the determination of TP, spikelets with mature anthers (about to open) were collected and processed. Briefly, six anthers per spikelet were dissected using a fine dissecting needle, after which 1.5 mL of AF6 buffer (Amphasys AG, Switzerland) was added and subsequently filtered using a 100 μm filter (Amphasys AG, Switzerland). The filtrate was then read using AmphaChip E00913, a 250 μm chip (Amphasys AG, Switzerland). Thresholds clearly separating pollen from debris was manually determined using the data from warm set-up and was subsequently used throughout the two experiments. TP was calculated by adding the upper left and upper right readings in the quadrant. NDA and POS were processed following methods described by Susanti et al. (2019) with some modifications. Shortly after floret opening, spikelets were collected from the middle of the main stem panicle. To determine POS, the stigma was dissected from the spikelets and the associated pollen grains were stained using a Lugol solution (Merck Pty. Ltd., Germany), and the number of fertile (engorged; stained black) were counted. The total NDA was counted out of the six anthers and along with POS, were visualized under a stereomicroscope (Olympus SZX10; Olympus Corporation, Japan). Using SS determined from Experiment 2 and this experiment, genotypes were then grouped into tolerant and sensitive RILs to facilitate statistical comparisons among the traits collected above.

### Statistical Analysis

Experiments were designed using the DiGGER package in R (Coombes, 2009). Statistical analysis was carried out on phenotypic traits collected from the full population (Experiments 1–4) using ASReml package in R (VSNi, United Kingdom). Best linear unbiased predictors (BLUPs) and heritabilities (Smith et al., 2006) of traits were computed using a model with genotype as a random effect. For the differential set experiment (Experiments 5 and 6), best linear unbiased estimators (BLUEs) were computed on a resolvable row-column design using a mixed linear model treating genotype as a fixed effect using the lme4 package in R (Bates et al., 2015). Testing for significance of difference between groups was also carried out using multcomp package in R (Hothorn et al., 2008). Significant genotypic variation among genotypes and groups tested were considered at *p* < 0.05, unless stated otherwise. Correlation between significant traits were determined through Pearson product-moment correlation coefficient using BLUPs and BLUEs computed in respective experiments.

### Quantitative Trait Loci Mapping Using Diversity Arrays Technology Sequence Data

Out of the 262 RILs, 252 were genotyped for single nucleotide polymorphism (SNP) using the Diversity Arrays Technology (DArT) genotyping-by-sequencing platform (DArTSeq). DArTSeq allowed for complexity reduction and sequencing of low copy sequences (corresponding predominantly to active genes) through the use of methylation sensitive restriction enzymes. These libraries were then sequenced using a next generation sequencing platform (Illumina HiSeq 2500) and the resulting sequences were aligned to *Oryza sativa* v7 reference genome to identify SNPs. DArTSeq resulted in a total of 6,508 SNP loci distributed across the rice genome and a total of 2,632 high quality polymorphic SNP loci were retained (Vinarao et al., 2021b) after filtering for call rates and markers following the expected allele frequency for RILs. Using the final genetic map and genotype data generated by Vinarao et al. (2021b) from the same population, a multiple QTL model (MQM) analysis (Broman et al., 2003) was carried out to identify genomic regions associated with SS and DTH collected from the full population. The logarithm of odds (LOD) threshold used to report QTL were based on the results of 1,000 permutations at a 5% significance level. Finally, using the bayesint function, Bayesian credible 95% confidence interval surrounding the identified QTL peak were determined in R/qtl (Broman et al., 2003).

### Whole-Genome Re-sequencing Analysis Between Parents and Haplotype Analysis Using a Public Database

High-quality genomic DNA from Sherpa was extracted using a modified cetyltrimethylammonium bromide extraction method (Doyle and Doyle, 1987). DNA quality was checked using 1% agarose gel electrophoresis and NanoDrop™ 8000 Spectrophotometer (Thermo Fisher Scientific, Waltham, MA, United States). Extracted DNA was sent to Macrogen Inc., South Korea where DNA libraries were prepared and sequenced. Library preparation was carried out using TruSeq Nano DNA 350 (Illumina, United States) and validated for concentration and size distribution using Agilent TapeStation D1000 Screen Tape (Agilent, United States). The resulting libraries were then sequenced with Illumina NovaSeq 6000 (Illumina, United States) paired-end 150bp reads. Raw reads (paired-end reads of 90 bp) of IRAT109 were retrieved from the National Center for Biotechnology Information Sequence Read Archive (NCBI SRA: ERS470547) which was made publicly available through the 3K Rice Genotyping Project (3KRGP) (Mansueto et al., 2017).

Initial quality control analyses of the whole-genome re-sequencing (WGRS) were carried out using the FastQC tool (Andrews, 2010) and then Trimmomatic (Bolger et al., 2014) was used to remove Illumina adapter sequences, filter reads shorter than 36 bp, and to trim the start and end of the sequences having quality lower than 5 (Ignacio et al., 2021). The trimmed sequences were then aligned to the *Oryza sativa* MSU release 7 reference genome^2^ using the Burrows-Wheeler Alignment maximal exact matches sequence aligner (Li, 2013), and were subsequently sorted and assigned respective groups. The resulting alignments were marked for PCR and optical duplicates and filtered for mapping quality (MAPQ) score greater than 20 (Li et al., 2009). Additionally, alignments with the following flag bits were retained: (1) read is paired and (2) read is mapped in a proper pair, and subsequent alignments were left realigned through the BamLeftAlign tool to homogenize positional distribution of insertions and deletions (indels). A bayesian genetic variant detection was carried out using FreeBayes to find SNPs, indels, multi-nucleotide polymorphisms and complex variants in a simple diploid calling manner with filtering and coverage: (1) MAPQ of 30, (2) minimum base quality of 20, and (3) minimum coverage of 15 to process a site (Garrison and Marth, 2012). Finally, variants were annotated and effect predicted using SnpEff tool (Cingolani et al., 2012). Variants were further filtered for polymorphism between parents using Trait Analysis by aSSociation, Evolution and Linkage software (Bradbury et al., 2007). Co-expression analysis for LOC_Os03g03100 was carried out using the online Rice Annotation Project Database (RAPDB) (Sakai et al., 2013) and gene ontology (GO) enrichment analysis was performed in RiceFREND (Sato et al., 2013). A built-in tool of the SNP-Seek database (Mansueto et al., 2017) was used to carry out haplotype analysis for LOC_Os03g03100 in the 3024 sequenced lines available through the 3KRGP. The Calinski criteria (Caliński and Harabasz, 1974) for automatic k-group determination was employed to cluster the lines into groups/haplotypes (Abbai et al., 2019). The All 3K SNP dataset containing 32 million SNPs was utilized.

## Results

### Phenotypic Characterization of the Full Population: Experiments 1 to 4

**FIGURE 1:**
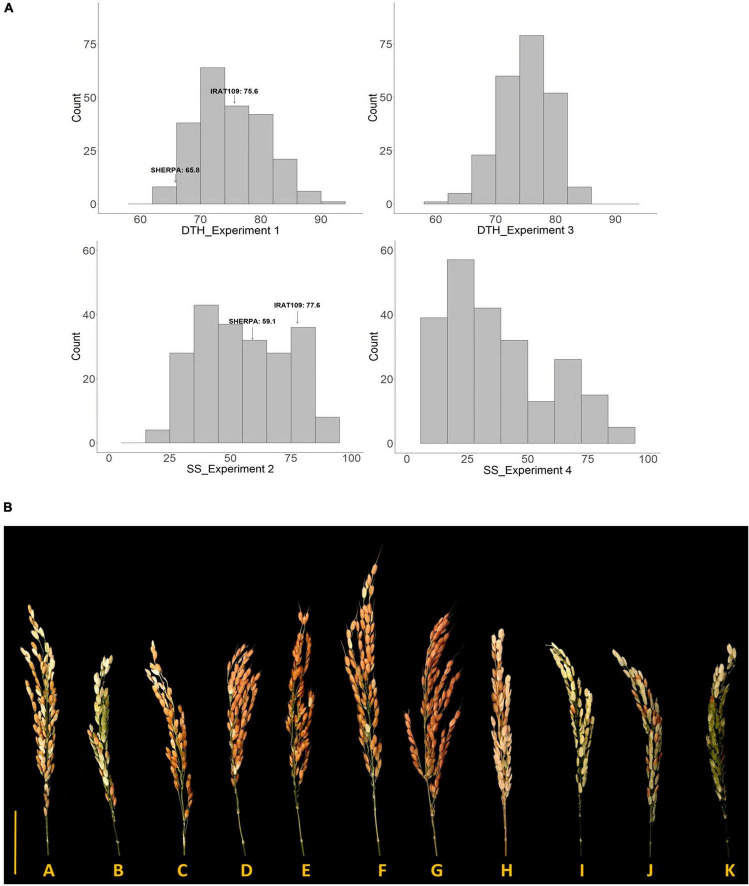
**(A)** Distribution of SS and DTH obtained from Sherpa/IRAT109 recombinant inbred lines in warm (Experiments 1 and 3) and cold (Experiments 2 and 4) conditions. SS, spikelet sterility; DTH, days to heading. **(B)** Variation in the response of differential rice recombinant inbred lines (RILs) derived from Sherpa/IRAT109 along with check genotypes exposed to 14 days low temperature (20/15°C, day/night) at young microspore stage. A—Sherpa; B—IRAT109; C—Norin PL8; D–K—recombinant inbred lines derived from Sherpa/IRAT109; Scale bar = 5 cm.

In Experiment 1, 244 RILs established and were subsequently used as indicators for determining the time of transfer of the genotypes to LT glasshouse in Experiment 2. Highly significant genotypic variation (*p* < 0.0001) was observed for all the phenotypic traits collected in Experiment 2 with heritabilities ranging from 0.67 to 0.86 (Table 1). Ten genotypes were found to escape the LT treatment and as such a total of 234 RILs were included in the following further analysis. Lijiangheigu and RL11 had relatively low SS, with 46.7 and 21.6%, respectively. In terms of the parents, Sherpa produced a lower SS, compared to IRAT109, with 59.1 and 77.6%, respectively. Within the RILs, 8.6% (21 genotypes) had SS from 0-30%, and therefore were considered LT tolerant, while the majority of the RILs (129, 52.9%) had SS greater than 50% (Figure 1A). The final tolerant selected RILs (19) had an average sterility of 27.2%, while the susceptible RILs (20) had an average of 82.1%. In terms of PH, Lijiangheigu was the tallest (117.7 cm) among the checks and RILs tested. IRAT109 was also taller (77.8 cm) than Sherpa (65.2 cm). The tallest derivative of Sherpa/IRAT109 recorded PH of 98.4 cm while the shortest was 52.9 cm. There was also a significant genotypic variation in SPP. IRAT109 showed a slightly higher SPP compared with Sherpa, with 78.4 and 66.5, respectively. The RILs had SPP from as high as 124.3 to as low as 43.9. In terms of DTH collected in Experiment 1, highly significant genotypic variation (*p* < 0.001) was also observed, with a heritability of 0.94. Of the parents, Sherpa, headed earlier (65.8 DAS) than IRAT109 (75.6 DAS), while their progenies took 64.6–90.5 days to head (Figure 1A). Correlation analysis among the RILs revealed significant relationships among the traits collected. Earlier heading genotypes in Experiment 1 tended to have higher sterility in Experiment 2 (*r* = −0.34^**^). Similarly, taller plants also tended to have lower SS (*r* = −0.24^**^).

**TABLE 1:** Percent spikelet sterility, plant height, spikelet number per panicle (Experiments 2 and 4), and days to the heading of the warm set (Experiments 1 and 3) of recombinant inbred lines derived from Sherpa/IRAT109 evaluated for low-temperature stress tolerance at young microspore stage.

	Expt 1	Expt 2			Expt 3	Expt 4		
	DTH	SS	PH	SPP	DTH	SS	PH	SPP
	RILs							
Min	64.6	19.2	52.9	43.9	59.5	6.9	55.8	56.7
Max	90.5	88.6	98.4	124.3	83.3	89.2	104	109.9
Mean	75.3**	54.7**	74.1**	78.8**	74.9**	37.9**	81.1**	80.1**
Heritability	0.94	0.72	0.86	0.67	0.73	0.81	0.72	0.47
	Parents							
Sherpa	65.8	59.1	65.2	66.5	ND	ND	ND	ND
IRAT109	75.6	77.6	77.8	78.4	ND	ND	ND	ND
	RIL Groups							
Tolerant RILs (19)	77.9	27.2	77.5	72.8	76.7	27.1	83.7	76.8
Sensitive RILs (20)	75.2	82.1	75.0	82.1	74.7	51.2	83.9	81.8

DTH, days to heading; PH, plant height (cm); SPP, number of spikelet per panicle; SS, percent spikelet sterility; ND, no data. ** p < 0.001.

Highly significant genotypic variation (*p* < 0.0001) was also observed for DTH and SS collected in Experiment 3 and 4 with heritabilities of 0.73 and 0.81, respectively (Table 1). DTH in Experiment 3 showed an average of 74.9 days with the top 25 earliest RILs showing an average DTH of 66.9 while the top 25 latest RILs registered 81.6 days (Figure 1A). In terms of SS from Experiment 4, the average was 37.9% with the tolerant RILs producing an average SS of 27.1% while the sensitive RILs had 51.2%. SS between Experiments 2 and 4 were also significantly correlated (*r* = 0.42^**^).

### Variation in Floral and Agronomic Traits From the Warm Set: Experiment 5

Highly significant genotypic variation (*p* < 0.001) was observed in the floral and agronomic traits collected in Experiment 5, except for NDA (Table 2). Heritabilities of the traits ranged from 0.67 (TP) to 0.96 (DTH). SS ranged from 0.7 to 25.7% with a mean of 5.8%. Comparing the parents, consistent with the full population evaluation, Sherpa headed earlier (66.4 days) than IRAT109 (71.6 days). In terms of the analysis of group effect, no significant difference was observed between the tolerant and sensitive RILs when grown under warm conditions—the groups had similar SS, were similar in days to heading, with similar floral trait results.

**TABLE 2:** Percent spikelet sterility, floral, and agronomic traits collected from the differential set experiment grown in warm conditions for the duration of the experiment (Experiment 5) and exposed to low temperature at young microspore stage (Experiment 6), using 51 RILs derived from Sherpa/IRAT109 and checks.

Genotype	SS	DTH	PH	FS	SPP	NDA	POS	TP
	**Experiment 5**							
Tolerant RILs (19)	5.1	73.6	98.8	86	90	NS	154	2890
Sensitive RILs (20)	6.8	71.5	97.5	89	95	NS	112	2721
Sherpa	3.0	66.4	88.7	111	115	NS	98	3037
IRAT109	7.9	71.6	104.3	72	78	NS	55	3094
Lijiangheigu	9.4	70.1	131.2	89	98	NS	120	1772
Norin PL8	1.8	54.8	106.0	88	90	NS	56	4365
RL11	0.7	62.7	92.8	52	52	NS	57	1726
Reiziq	8.2	67.5	97.7	83	90	NS	93	3110
RILs (ALL)	5.6	72.1	97.4	87	92	NS	114	2842
Heritability	0.77	0.96	0.95	0.88	0.88	NS	0.68	0.67
	**Experiment 6**							
Tolerant RILs (19)	18.0**	80.3	82.8	57**	69	5.4**	92**	2091**
Sensitive RILs (20)	73.6	75.6	81.7	21	79	2.8	42	1213
Sherpa	50.8	71.0	76.0	51	104	2.8	42	1032
IRAT109	69.9	79.3	86.3	14	62	3.3	20	1096
Lijiangheigu	17.9	83	117.7	66	81	5.2	33	2555
Norin PL8	16.4	60.1	82.7	48	56	3.7	57	1077
RL11	9.1	78.1	86.4	36	40	6.0	121	2188
Reiziq	92.9	75.0	82.2	4	59	1.3	16	472
RILs (ALL)	51.0	77.5	81.1	35	71	3.7	59	1572
Heritability	0.91	0.97	0.93	0.86	0.77	0.71	0.62	0.86

SS, percent spikelet sterility; DTH, days to heading; PH, plant height (cm); FS, number of filled spikelets; SPP, number of spikelets per panicle; NDA, number of dehisced anthers; POS, number of filled pollen on stigma; TP, total pollen per spikelet; NS, no significant difference among genotypes. **Significantly different compared with sensitive RILs at p < 0.01.

### Floral Traits Associated With LT Tolerance at YMS: Experiment 6

Highly significant genotypic variation (*p* < 0.001) was observed in the floral traits (NDA, POS, and TP) as well as the key agronomic traits [SS (Figure 1B), DTH, PH, and FS], when grown under LT in Experiment 6 with heritabilities ranging from 0.62 (POS) to 0.97 (DTH in LT experiment) (Table 2). RL11, Norin PL8, and Lijiangheigu had low SS with 9.1, 16.6, and 18.0%, respectively. Sherpa consistently produced lower SS (50.8%) compared with IRAT109 (69.9%). Their derived RILs showed an average of 51.0% SS, with genotypes as low as 2.3% to as high as 99.6%. Examining the difference between RIL genotype groups, tolerant RILs produced significantly lower SS with an average of 18.0% compared with the sensitive RILs (73.6%). Tolerant RILs showed significantly higher number of FS, more than double compared to the sensitive RILs. Analysis into the floral traits between these groups also indicated that tolerant RILs had significantly higher NDA, with an average 2.6 more dehisced anthers and an increased number of POS compared with sensitive RILs (92 vs 42, 119%). Furthermore, tolerant RILs had 72% more pollen per spikelet than the sensitive RILs. It is worth noting that there was no significant difference between DTH of tolerant and sensitive RIL groups owing to the stratified sampling strategy employed during genotype selection. In summary, after exposure to LT conditions at YMS, tolerant RILs had lower SS, produced higher NDA, POS, and TP compared to sensitive RILs. TP between Experiments 5 and 6 were related (*r* = 0.4^**^), indicating that genotypes with high number of pollen per spikelet in warm conditions also tended to have higher TP in LT.

### Genomic Regions Associated With LT Tolerance and DTH

**FIGURE 2:**
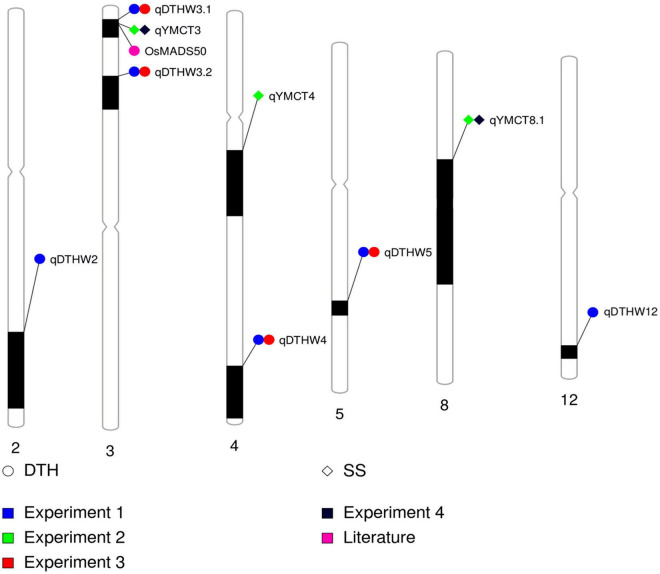
Graphical summary and physical locations of QTL identified to be associated with DTH and SS across four experiments conducted. Physical map positions are based on the Nipponbare sequence at RAP database. DTH-days to heading; SS-spikelet sterility.

**FIGURE 3:**
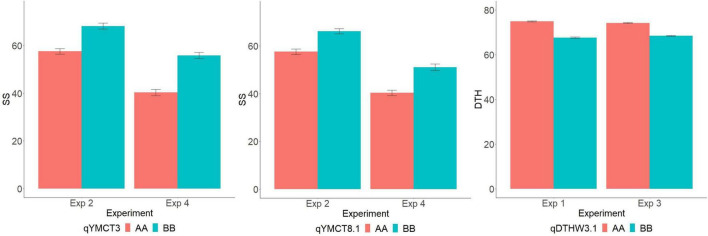
Allelic effect (AA—Sherpa, BB—IRAT109) *of qYMCT3* and *qYMCT8.1* on spikelet sterility (SS) of RILs in Experiments 2 and 4, and of *qDTHW3.1* on days to heading (DTH) in Experiments 1 and 3.

Of the 252 RILs with SNP genotype information, 241, 228, 214, and 229 RILs were used to map genomic regions associated with DTH in Experiment 1 and 3, and SS in Experiment 2 and 4, respectively. A total of ten RILs were determined to have escaped the LT treatment in Experiment 2 and were therefore excluded from the QTL mapping effort for SS. Using the corresponding SNP genotyping data and phenotypic data from the full population, linkage mapping was carried out and identified a total of nine genomic regions: three associated with SS while six were for DTH (Table 3, Figure 2, and Supplementary Figure 1). MQM analysis was carried out for SS with a LOD threshold of 3.06 and 3.12 and subsequently identified three and two genomic regions associated with lower SS for Experiments 2 and 4 respectively, explaining 19.5–22.4% of the total genotypic variation. Of the QTL identified, two (*qYMCT3* and *qYMCT8.1*) were identified in both experiments while one (*qYMCT4*) was identified only in Experiment 2. *qYMCT3* with an LOD score of 4.14–6.96 was located around 2.19 Mb physical position, with a confidence interval of 0.9–11.7 cM. Introgression of this QTL with the Sherpa (AA) allele was estimated to lower the SS of a genotype by 10.6–15.4% (Figure 3) and this region explained about 7.24–12.08% of the genotypic variation observed in the population. *qYMCT8.1* with a LOD score of 3.07–3.26 was located around 18.45 Mb physical position of Chromosome 8. Introgression of the Sherpa allele from *qYMCT8.1* was estimated to decrease SS of a genotype by 8.6–10.8% (Figure 3). The SS and SNP genotype composition of three selected RILs harboring the favorable (AA) allele of *qYMCT3* and *qYMCT8.1* (Supplementary Figure 2) were shown in Table 4 in contrast to the composition of one RIL harboring the unfavorable allele. Additionally, *qYMCT4* was identified on Chromosome 4 (14.89 Mb) with a LOD score of 3.77 (Figure 2). In *qYMCT4* the introgression of the IRAT109 (BB) allele decreased SS by about 9.6%.

**TABLE 3:** Genomic regions identified to be associated with lower spikelet sterility at young microspore stage and days to heading from Sherpa/IRAT109 RIL population across different experiments.

Experiment	Trait	QTL	Chr	Peak Marker	Mb	cM	CI (cM)	LOD	AE	R^2^
Exp 2	SS	*qYMCT3*	3	Chr3_2188710	2.19	6.0	0.9–11.7	4.14	5.3	7.24
		*qYMCT4*	4	Chr4_14885422	14.89	28.2	22.5–36.5	3.77	–4.8	6.55
		*qYMCT8.1*	8	Chr8_18453647	18.45	62.7	51.1–96.9	3.07	4.3	5.29
										(22.43)
Exp 4	SS	*qYMCT3*	3	Chr3_2188710	2.19	7.6	2.7–8.22	6.96	7.7	12.08
		*qYMCT8.1*	8	Chr8_18453647	18.45	64.0	54.4–105.6	3.26	5.4	5.46
										(19.53)
Exp 1	DTH	*qDTHW2*	2	Chr2_28954645	28.95	128.0	121.5–156.6	3.75	1.0	2.74
		*qDTHW3.1*	3	Chr3_1248074	1.25	2.0	0.9–7.6	41.58	–3.7	44.72
		*qDTHW3.2*	3	Chr3_5909043	5.91	25.2	16.3–42.1	6.44	1.3	4.83
		*qDTHW4*	4	Chr4_29775386	29.78	98.0	92.1–122.9	7.25	1.0	5.47
		*qDTHW5*	5	Chr5_26356516	26.36	104.0	93.9–106.5	13.69	1.9	11.02
		*qDTHW12*	12	Chr12_25487755	25.49	102.0	97.8–107.5	6.45	1.3	4.83
										(63.14)
Exp 3	DTH	*qDTHW3.1*	3	Chr3_1248074	1.25	2.0	0.9–2.7	23.93	–2.9	32.70
		*qDTHW3.2*	3	Chr3_5909043	5.91	30.0	25.0–42.1	11.32	2.3	13.51
		*qDTHW4*	4	Chr4_30700814	30.7	112.0	105.5–122.9	9.54	1.5	11.18
		*qDTHW5*	5	Chr5_23611047	23.61	96.0	93.2–100.8	10.21	1.7	12.05
										(47.39)

*Mb, physical map position of each marker based on the Nipponbare sequence at RAP database (http://rapdb.dna.affrc.go.jp/); CI, bayesian credible confidence intervals in centiMorgan (cM); AE, additive effect of the allele from IRAT109 compared with that from the donor genotype, Sherpa; R^2^, the percentage of the genotypic variance explained by each QTL (quantitative trait locus). Numbers in parentheses indicate the percentage of the variance explained by multiple QTL models. SS, percent spikelet sterility; DTH, days to heading; Exp, Experiment; LOD, logarithm of odds.*

**TABLE 4:** Spikelet sterility (SS,%) from Experiments (Exp) 2 and 4 of selected recombinant inbred lines (RILs) and parents along with their respective allelic composition for *qYMCT3* and *qYMCT8.1*.

Parent/RIL	*qYMCT3*	*qYMCT8.1*	SS		
			Exp 2	Exp 4	Mean
SHIR602497	AA	AA	26.7	8.1	17.4
SHIR602542	AA	AA	29.5	17.1	23.3
SHIR603103	AA	AA	29.6	13.6	21.6
SHIR602522	BB	BB	86.4	76.1	81.2
Sherpa	AA	AA	59.1	ND	59.1
IRAT109	BB	BB	77.6	ND	77.6

*ND, no data.*

As initial phenotypic analyses indicated the possible crosstalk between SS and DTH, MQM analysis was conducted and identified genomic regions associated with DTH in warm conditions, with LOD greater than the 3.09 and 3.08 computed threshold for Experiments 1 and 3, respectively. A total of six genomic regions located in five chromosomes (2, 3, 4, 5, and 12) were identified to influence DTH (Table 3, Figure 2, and Supplementary Figure 1). The final QTL model for Experiments 1 and 3 were able to explain 47.4–63.1% of the variation in DTH. In both experiments, *qDTHW3.1* located between genetic distance of 0.9–7.6 cM was the major QTL identified in this population with a LOD score of 23.93–41.58 and alone was able to explain 32.7–44.7% of the genotypic variation. Interestingly, this QTL was shown to co-locate with *qYMCT3* and the analysis also showed that introgression of the AA allele resulted in the genotypes to reach heading about 5.7–7.4 days (Figure 3) later compared to genotypes with BB allele. Of the five remaining QTL, three (*qDTHW3.2, qDTHW4*, and *qDTHW5*) were identified in both experiments while *qDTHW2* and *qDTHW12* were identified only in Experiment 1. The next major genomic region associated with DTH was found in chromosome 5 (*qDTHW5*) with a LOD score of 10.21–13.69 and an *R*^2^ of 11–12.1%. Introgression of the Sherpa allele was shown to result in an earlier heading of the genotypes tested, up to about 3.8 days. *qDTHW3.2* had a LOD score of 6.44–11.32 and explained 4.8–13.5% of the variation. Introgression of the Sherpa allele from *qDTHW3.2* also resulted in an estimated 2.6–4.5 earlier DTH. The other remaining QTL (*qDTHW2, qDTHW4* and *qDTHW12*) showed modest effects with LOD scores ranging from 3.75 to 9.54 and were able to explain between 2.7 and 11.2% of the variation. Similar to *qDTHW5* and *qDTHW3.2*, introgression of the Sherpa allele from these QTL resulted in earlier heading with effects ranging from 2 3 days earlier than with IRAT109 allele. Significant interaction between *qDTHW3.1* and *qDTHW4* was found in Experiment 1 with a LOD score of 3.05 and explained 2.2% of the variation. Similarly, in Experiment 3, a significant epistatic interaction between *qDTHW3.2* and *qDTHW4* was also detected, with a LOD score of 3.72 and explaining 4.1% of the variation. Looking into the additive effects of the genomic regions identified, although *qDTHW3.1* had the strongest effect (Sherpa allele cause delayed heading), the magnitude of the five QTL outweighed the effect of *qDTHW3.1*.

Using the MSU Rice Genome Annotation Project (Kawahara et al., 2013), a survey of previously identified genes associated with DTH along with the *qDTHW3.1* confidence interval was carried out. It was found that two cloned genes associated with heading in rice: *Ehd4* (LOC_Os03g02160, Chr03:717447-720837) and *DTH3* (LOC_Os03g03100, Chr03: 1298070-1300384) were located within this region.

### Comparative Sequence Analysis Between Parents

Analysis of the whole genome sequence data obtained from IRAT109 and Sherpa showed more than 109 and 69 million reads passed QC for the genotypes, respectively (Supplementary Table 1). Of these reads, a high percentage of the sequences were mapped with the reference Nipponbare genome at 95.8 and 98.2%, respectively for IRAT109 and Sherpa. After filtering for duplicate reads and retaining properly paired reads, the depth of coverage was calculated to be around 22X and 21X, for IRAT109 and Sherpa, respectively.

Variant calling carried out using Nipponbare reference sequence identified a total of 1,094,121 variants, of which 79.8, 7.65, 5.14, and 5.68%, were SNPs, multiple nucleotide polymorphisms (MNPs), insertions and deletions, respectively (Supplementary Table 2). Chromosome wise, chromosome 1 had the highest number of variants with 132,376 while chromosome 9 showed the lowest with 45,610. On average, the frequency of variant detection occurred every 342 number of bases. In terms of the number of variants by type and region, majority of the variants occurred in the intergenic region, followed by upstream and downstream gene variants, respectively. Finally, looking into the functional class of the variants, 55.6% of the variants were predicted to cause missense amino acid change in the protein product while 43.18 and 1.18% were predicted to cause silent and non-sense effects.

**FIGURE 4:**
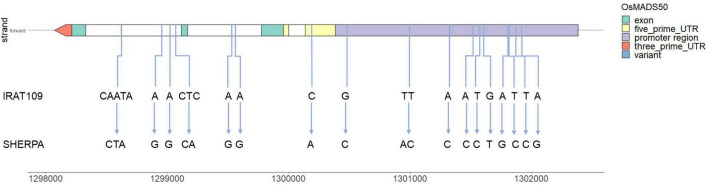
Variants identified in LOC_Os03g03100 (*OsMADS50*) gene between Sherpa and IRAT109 and their relative positions across the gene model.

Using the variants identified, sequence variation between the parents in the two DTH genes identified to co-locate with the *qDTHW3.1/qYMCT3* locus was investigated. It was found that no polymorphism occurred between Sherpa and IRAT109 in LOC_Os03g02160 while a total of 17 variants were identified in LOC_Os03g03100 gene (Figure 4 and Supplementary Table 3). Of the variants detected, 14 (82%) were SNPs while two were predicted to be complex variants and a single insertion variant. In terms of their effects, 10 were predicted to be upstream gene variants, one 5′ UTR variant, and the rest (six) were intron variants. Co-expression network analysis (hierarchy: 3, MR rank: 3) was carried out in RAPDB for LOC_Os03g03100 (RAPDB ID: Os03g0122600), and a total of 17 genes (Supplementary Table 4). Gene ontology (GO) enrichment analysis using these genes showed significant GO molecular functions (Supplementary Figure 3) related with calcium ion transporter activity (GO:0005388 and GO:0015085) as well as ATPase activity coupled with transmembrane movement of ions and substances (GO:0015662, GO:0042625, and GO:0042626). Haplotype analysis showed the presence of three haplotypes in LOC_Os03g03100 (*OsMADS50*, Supplementary Figure 4). It was noted that haplotype 2 (pink group) had the highest number of accessions, with 66.5%, followed by haplotype 1 (brown group) with 18.0% and finally by haplotype 3 (blue group) with 15.5%. Nipponbare and Sherpa clustered with haplotype 3 along with 60.4% of temperate japonica (tempjap) present in the 3KRGP, while haplotype 1 had 39.2% of the tempjaps (as well as IRAT109) and haplotype 2 only had one tempjap accession.

## Discussion

### Genotypic Variation in SS and Identification of Promising RILs Derived From Sherpa/IRAT109

The current paper evaluated RILs derived from the LT tolerant Australian rice variety, Sherpa for variation in SS and other key traits in controlled temperature glasshouse conditions. Evaluation of the full set of RILs resulted in a mean SS of 54.7 and 37.9% from Experiments 2 and 4, respectively and similarly, using a set of differential genotypes revealed a mean SS of 51.0% (Experiment 6). Across the two full population evaluations, a total of 13 RILs were identified to perform well with mean SS below 30%. The mean SS from our current experiments were comparable with previous results (Susanti et al., 2019) using the similar screening method developed by Mitchell et al. (2016). Likewise, the heritability values of SS from the experiments conducted in this study ranged from 0.72 to 0.90 and were also similar to a previous report (Susanti et al., 2019). This consistently high heritability of the trait along with the existence of large genetic variation (Guo et al., 2020) indicates that SS may have a good response to selection. Additionally, Ha et al. (2018) were able to show consistency in low-temperature tolerance between AP and PW indicating that current results can be utilized in both growing conditions. To facilitate the efficient development of these genotypes, it is imperative to identify genomic regions controlling SS for their use in molecular marker-assisted breeding programs.

### Key Floral Traits Linked to Low SS

To our knowledge, for the first time, the current study has shown the utility of an impedance flow cytometer in measuring TP of rice both in the context of ideal and low-temperature conditions. Additionally, tolerant genotypes with low SS showed higher NDA and TP, indicating that these two floral traits may be used as surrogates to select for genotypes with low SS in low-temperature conditions. NDA is relatively easy to phenotype and would require a simple stereomicroscope while TP measured using a flow cytometer (Heidmann et al., 2016) can be collected in minutes, both several weeks before the data for SS after panicle maturity becomes available. We utilized a set of differential genotypes to dissect the relationships between floral traits and the development of low SS. In the experiment where genotypes were exposed to LT, it was shown that the low SS tolerant group developed a higher number of pollen per spikelet resulting in higher NDA and eventually higher POS. The importance of pollen number in anther in determining SS after exposure to LT conditions has been demonstrated in previous studies (Mitchell et al., 2016; Susanti et al., 2019) and was supported by the fact that pollen swelling was found to be the prime force for anther dehiscence (Matsui et al., 1999). It is important to note though that this relationship with SS was absent in warm ideal growing conditions indicating that the previous relationships observed were only induced after exposure to LT conditions. Farrell et al. (2006b) also noted that SS was related to a number of engorged pollen and another length only after cold water exposure. Significant correlation for TP between warm and LT conditions also existed although there was no significant association between TP from warm and SS of LT experiment. This suggested that the genotypic variation in TP in warm conditions affected the variation in TP in LT treatment to some extent but was not strong enough to affect SS in LT conditions and ultimately, LT tolerance.

### Genomic Regions Associated With LT Tolerance at YMS

Three genomic regions controlling LT tolerance at YMS were identified, located at chromosomes 3 (*qYMCT3*), 8 (*qYMCT8*), and 4 (*qYMCT4*), and were found to co-locate with previously reported QTL. Several genomic regions associated with LT tolerance at various stages of crop development have been previously identified. In terms of the reproductive stage, a few regions have been identified to be associated with tolerance at either YMS or the booting stage (Ye et al., 2010; Lei et al., 2018; Proud et al., 2022). QTL *qYMCT3* was found to be located in the short arm of chromosome 3 roughly translating to between 1.25 and 2.98 Mb physical map position. This QTL was found to co-locate with *qCTSF3.1*, a genomic region associated with LT tolerance at the booting stage identified using chromosome segment substitution lines derived from a cross between *japonica* and *indica* varieties (Lei et al., 2018). By utilizing a genome-wide association mapping approach, Guo et al. (2020) identified SNPs located in 15.87–19.49 Mb of chromosome 8 using 522 diverse cultivars. Finally, the confidence interval of *qYMCT4* was also found to co-locate with a genomic region identified by examining 168 diverse accessions in high altitudes (Guo et al., 2020). *qYMCT4* was identified only in Experiment 2 and may suggest that this region is significant in cooler/harsher temperatures. A similar observation was made in DTH QTL identified which may signify the effect of these genomic regions in the short days (Experiment 3), in long day/natural light conditions (Experiment 1), or both. Identification of these inherent useful genomic regions from Sherpa should be helpful in (1) identifying existing varieties with favorable allele or QTL combinations and (2) introgressing additional LT tolerance genes/QTL to further improve the tolerance levels of the variety or subsequent development of improved genotypes with multiple LT tolerance QTL. Pyramiding of favorable alleles of high-value genomic regions and cloned genes (*CTB4a, Ctb1, bZIP73*, and *OsAPX1*) have been shown to increase LT tolerance of genotypes in North China (Guo et al., 2020). Transgressive segregation in both DTH and SS existed. Specifically, in terms of SS, both parents contributed genomic regions and alleles associated with low SS, which may explain the transgressive segregation observed in this population. Although rare, transgressive segregation for SS after LT exposure at YMS or booting stage has been previously observed (Takeuchi et al., 2001). The final QTL model for SS in the current study explained around a quarter of the phenotypic variation in the population. The construction of specialized mapping populations such as chromosome segment substitution lines may aid in the identification of additional genomic regions governing SS (Yamamoto et al., 2009).

There is evidence of a pleiotropic effect of the QTL/gene controlling SS for LT tolerance (*qYMCT3*) and DTH (*qDTHW3.1*) in this population. To our knowledge, our subsequent analyses are the first attempt in linking *OsMADS50* with LT tolerance in rice. Several efforts using genomics, transcriptomics, and gene functional analyses conducted in rice and *Arabidopsis thaliana* were in congruence with the observations made in our current study. Using a set of genotypes derived from the IRRI breeding program, *qDTHW3.1* was also found to be associated with DTH in the indica subpopulation (Begum et al., 2015). The same region was also identified using a NIL derived from *Oryza glaberrima* (Bian et al., 2011). Additionally, it was pointed out that LOC_Os03g03100 was the best candidate for this region (Begum et al., 2015). WGRS comparative analysis in our current study supported this claim by showing the absence of polymorphism between parents in *Ehd4*, while several polymorphisms were identified in LOC_Os03g03100. The primary candidate gene for *qYMCT3*/*qDTHW3.1* was therefore LOC_Os03g03100 encoding for a MADS-box transcription factor (*OsMADS50*). Functional analysis of *OsMADS50* showed that it is an ortholog of *A. thaliana’s SUPPRESSOR OF OVEREXPRESSION OF CO1/AGAMOUS-LIKE 20 (SOC1/AGL20)* (Lee et al., 2004). Their experiments also showed that overexpression of *OsMADS50* resulted in early flowering while RNAi and knock-out plants exhibited phenotypes with late flowering (Lee et al., 2004; Ryu et al., 2009). MADS-box gene family has been identified as important molecular players in abiotic stress responses such as LT (Castelan-Munoz et al., 2019). *SOC1* is a known key floral activator integrating multiple floral inductive pathways in *A. thaliana* and was in parallel to the observation in rice through its ortholog, *OsMADS50*. *SOC1* was also shown to participate in LT sensing. Loss of function analysis (*soc1-2*) showed induced expression of LT responsive genes such as cold regulated and C-repeat/dehydration response element binding factors, while its overexpression resulted in decreased expression of such genes, indicating that *SOC1* was a negative regulator of the expression of LT response genes (Seo et al., 2009). The above series of experiments demonstrated the crosstalk between flowering and LT sensing in *A. thaliana*, and with the shared genomic regions identified in the current study for DTH and SS, it may very well be the same case in rice, for *OsMADS50*. Co-expression and GO enrichment analyses of genes associated with the expression of *OsMADS50* showed its link with calcium ion transport. Cold signal transduction has also been shown to involve the activation of calcium ion channels and pumps; and increasing evidence shows that calcium signaling plays a crucial role in conferring LT tolerance among plants (Yuan et al., 2018), further strengthening the plausible crosstalk between flowering and LT tolerance as mediated by *OsMADS50*. However, further experimentation utilizing over-expression and knock-out mutants of *OsMADS50* evaluated in LT environments is needed to underpin this crosstalk and relationship in rice. Additionally, while the current analysis and literature point out *OsMADS50* as the most prudent candidate gene for *qYMCT3* and *qDTHW3.1*, it is still also possible that another gene or independent genes contribute to LT tolerance and DTH in this population.

With these genomic regions identified, their validation in multiple genetic backgrounds is an imperative step for their utility in respective breeding programs (Cobb et al., 2019). As the plausible useful allele *OsMADS50* may result in delayed heading, it is also important to identify additional genomic regions which may nullify this effect. Although late to head, *osmads50* mutant plants did eventually flower indicating that other positive regulators induced flowering in the absence of the expression of *OsMADS50* (Ryu et al., 2009). This is in congruence with our data which identified other genomic regions associated with earlier flowering from Sherpa. The presence of the other five genomic regions with Sherpa having the favorable (early flowering) allele suggested that other genomic regions may be used to produce LT tolerant, early flowering genotypes from this donor. Furthermore, haplotype analysis showed the possible utility of *OsMADS50* favorable haplotype across rice subpopulations. The Sherpa haplotype (haplotype 3) was shown to have a relatively high frequency (60%) in the tempjap subpopulation suggesting that although it might be useful to introgress this haplotype into genotypes with non-favorable haplotype, the utility of this gene to improve LT tolerance in tempjaps will be of higher magnitude in the survey and identification of genotypes already possessing this useful haplotype. This type of utility was highlighted recently in an elite breeding core panel where several high-value disease and insect resistance genes were present at considerable frequencies (although not fixed) and thus represent haplotypes/alleles that are easily selectable in existing breeding populations (Juma et al., 2021). After successful QTL validation, molecular markers could be designed for their survey and introgression in breeding programs (Vinarao et al., 2021a). An assessment of high throughput molecular markers in rice showed that only two QTL for seedling LT tolerance, *SCT1*, and *COLD1*, were developed for this purpose^3^ indicating the opportunity to develop markers for reproductive stage LT tolerance. Therefore, the development of high throughput molecular markers for the three QTL identified in this study as well as known cloned LT tolerance genes should aid in the tailored genotype identification and pyramiding of additional beneficial genomic regions to develop AP adapted, LT tolerant genotypes.

## Conclusion

We have identified three genomic regions in rice associated with low SS after exposure to LT at YMS. These may be surveyed in existing elite germplasm and stacked with other high-value LT tolerance genes to develop genotypes with high levels of LT tolerance during the reproductive stage, both in AP and in traditional flooded systems. *OsMADS50* was demonstrated as the best candidate gene for *qDTHW3.1* and *qYMCT3*, indicating the potential pleiotropic effect of the QTL/gene. The possible crosstalk of flowering and LT sensing in rice was also explored using this gene. Additionally, we have confirmed the importance of an increased number of developed pollen grains in the anther/spikelet, which in turn affects NDA and POS, and ultimately determines lower SS. NDA and TP may be used as surrogates for SS being conveniently measurable traits and can be phenotyped before SS becomes available. Validation of the effects of the QTL identified in this study using several genetic backgrounds and subsequent development of high throughput molecular markers should pave the way for the introgression of these genes and regions into target cultivars. Further work including functional analysis of the candidate gene in LT conditions as well as pyramiding of other high-value QTL for key traits—LT tolerance and deeper roots will enable breeding not only for AP adapted genotypes but also for the development of cultivars adapted to LT in temperate growing regions, and ultimately to produce climate-resilient rice.

## Data Availability Statement

The datasets presented in this study can be found in online repositories. The name of the repository and accession number can be found at: National Center for Biotechnology information (NCBI) BioProject, https://www.ncbi.nlm.nih.gov/bioproject/, PRJNA813905.

## Author Contributions

RV, CP, PS, SF, and JM: conceptualization and writing—review and editing. RV and CP: methodology, software, and formal analysis. RV: validation and writing—original draft preparation. CP, SF, and JM: supervision. JM: funding acquisition. All authors have read and agreed to the published version of the manuscript.

## Conflict of Interest

The authors declare that the research was conducted in the absence of any commercial or financial relationships that could be construed as a potential conflict of interest.

## Publisher’s Note

All claims expressed in this article are solely those of the authors and do not necessarily represent those of their affiliated organizations, or those of the publisher, the editors and the reviewers. Any product that may be evaluated in this article, or claim that may be made by its manufacturer, is not guaranteed or endorsed by the publisher.

## Footnotes

1 https://www.agrifutures.com.au/wp-content/uploads/publications/13-054.pdf, accessed on 10 October 2021.

2 https://phytozome-next.jgi.doe.gov/info/Osativa_v7_0, accessed on 10 October 2021.

3 https://isl.irri.org/services/genotyping/trait-based-genotyping, accessed on 10 October 2021.

## Supplementary Material

Supplementary Figure 1 LOD profiles for QTL associated with SS and DTH were detected using single and multiple QTL models. Red and green line—multiple QTL LOD profiles for SS for Experiments 2 and 4, respectively; black and blue line—multiple QTL model LOD profiles for DTH for Experiments 1 and 3. Dashed lines indicate LOD threshold levels used to identify QTL associated with the trait. The color of the dashed lines corresponds to the color of the LOD profiles for the specific analysis carried out. DTH, days to heading; LOD, logarithm of odds; SS, percent spikelet sterility; QTL, quantitative trait loci.

Supplementary Figure 2 Graphical SNP genotype composition of selected RILs harboring favorable (SHIR602497, SHIR602542, and SHIR603103) and unfavorable (SHIR602522) QTL alleles and their respective SS (mean of Experiments 2 and 4). Blue and red lines indicate Sherpa and IRAT109 alleles, respectively. Arrows indicate the presence of a favorable QTL allele.

Supplementary Figure 3 Gene ontology (GO) enrichment analysis using *OsMADS50* co-expressed genes.

Supplementary Figure 4 Haplotype analysis using SNPs identified in LOC_Os03g03100 (*OsMADS50*) using 3,024 sequenced genomes from the 3K Rice Genotyping Project.

Supplementary Table 1 Summary statistics for whole-genome sequence analysis of IRAT109 and Sherpa using Illumina NovaSeq 6000.

Supplementary Table 2 Variants identified using IRAT109 and Sherpa resequencing data per chromosome.

Supplementary Table 3 Variants identified in LOC_Os03g03100 (*OsMADS50*) gene between Sherpa and IRAT109.

Supplementary Table 4 Hierarchy, locus ID, and description of co-expressed genes with *OsMADS50*.
