# Supplementary figures and images for: Genomic Regions and Floral Traits Contributing to Low Temperature Tolerance at Young Microspore Stage in a Rice (Oryza sativa L.) Recombinant Inbred Line Population of Sherpa/IRAT109
Source: Front Plant Sci. 2022 Apr 29;13:873677. doi: 10.3389/fpls.2022.873677 (PMC9100824; doi:10.3389/fpls.2022.873677)

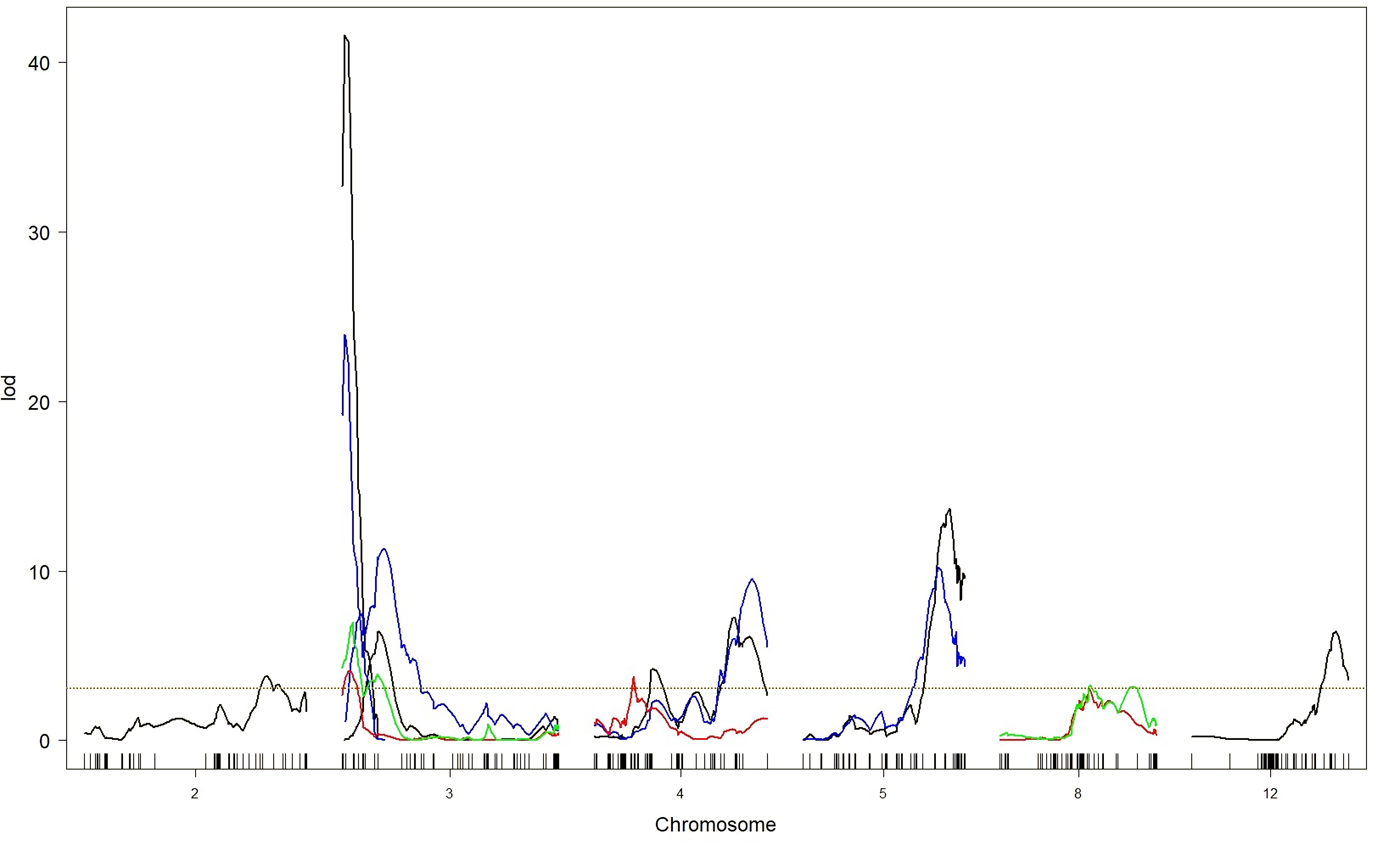

Supplement: Supplementary Figure 1 — LOD profiles for QTL associated with SS and DTH were detected using single and multiple QTL models. Red and green line—multiple QTL LOD profiles for SS for Experiments 2 and 4, respectively; black and blue line—multiple QTL model LOD profiles for DTH for Experiments 1 and 3. Dashed lines indicate LOD threshold levels used to identify QTL associated with the trait. The color of the dashed lines corresponds to the color of the LOD profiles for the specific analysis carried out. DTH, days to heading; LOD, logarithm of odds; SS, percent spikelet sterility; QTL, quantitative trait loci. [file Image_1.JPEG]

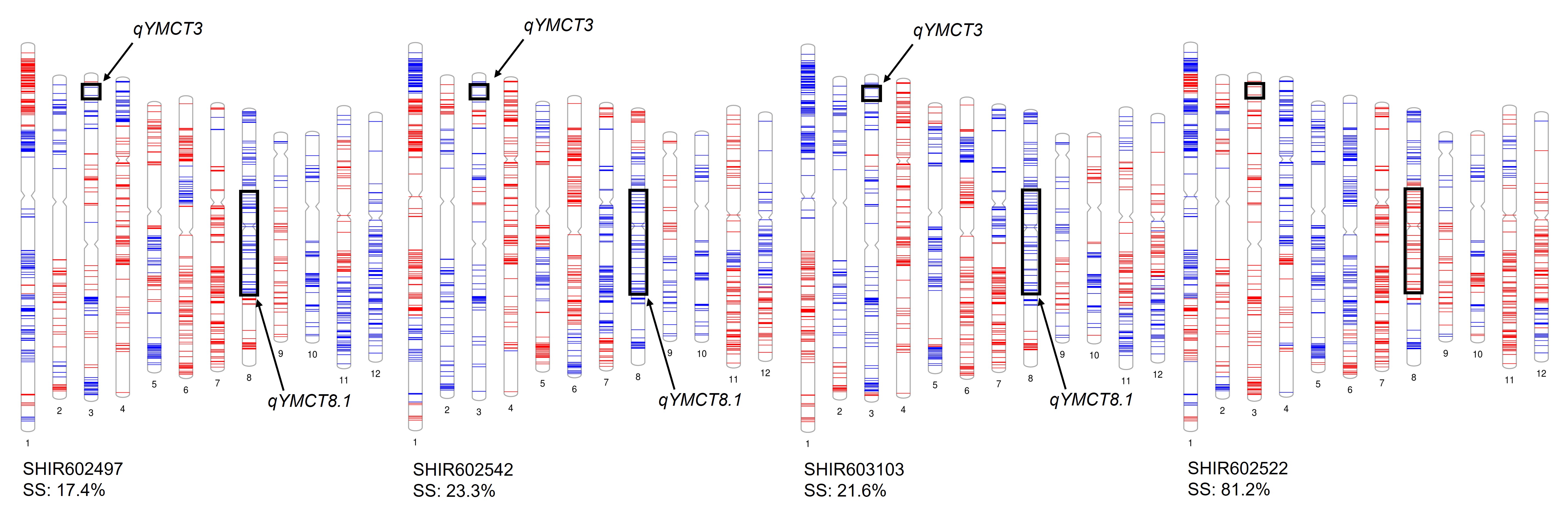

Supplement: Supplementary Figure 2 — Graphical SNP genotype composition of selected RILs harboring favorable (SHIR602497, SHIR602542, and SHIR603103) and unfavorable (SHIR602522) QTL alleles and their respective SS (mean of Experiments 2 and 4). Blue and red lines indicate Sherpa and IRAT109 alleles, respectively. Arrows indicate the presence of a favorable QTL allele. [file Image_2.JPEG]

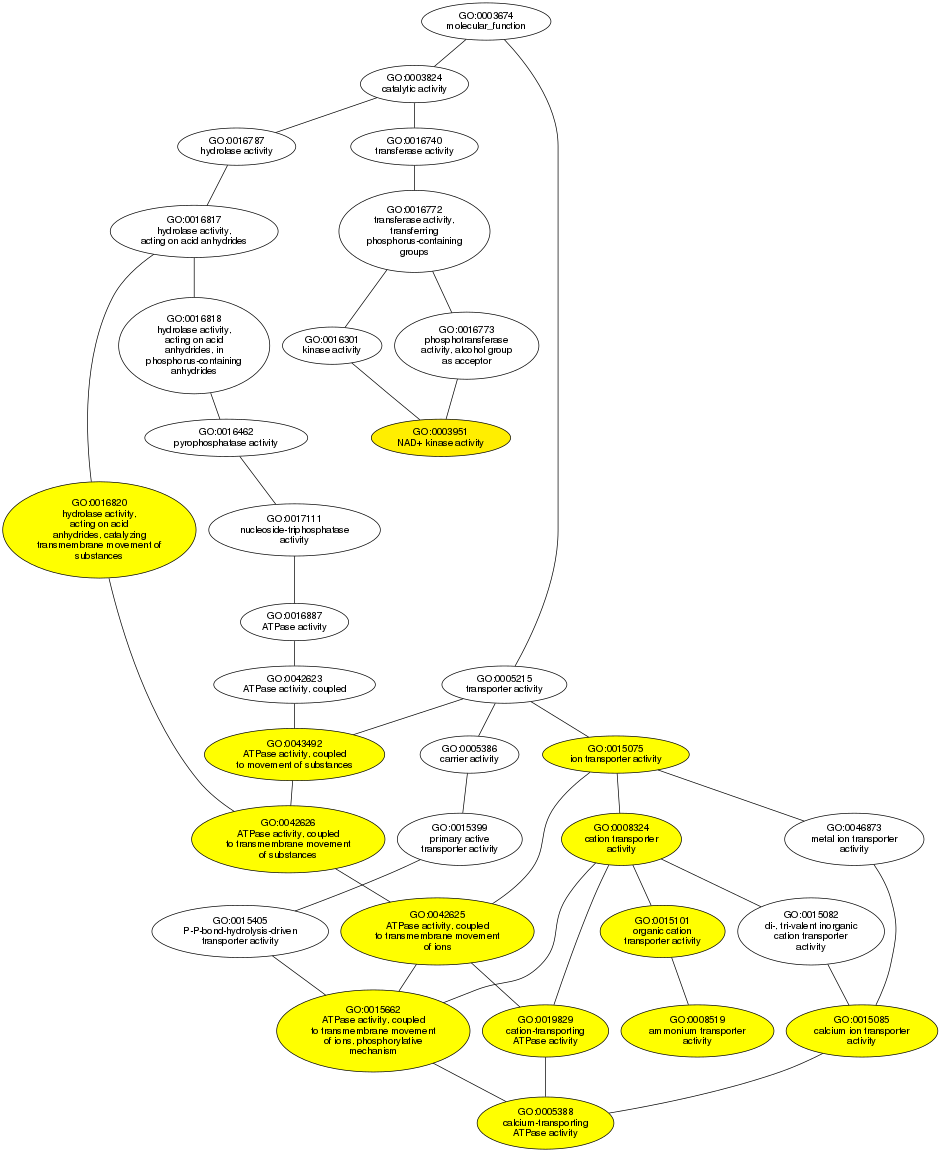

Supplement: Supplementary Figure 3 — Gene ontology (GO) enrichment analysis using OsMADS50 co-expressed genes. [file Image_3.PNG]

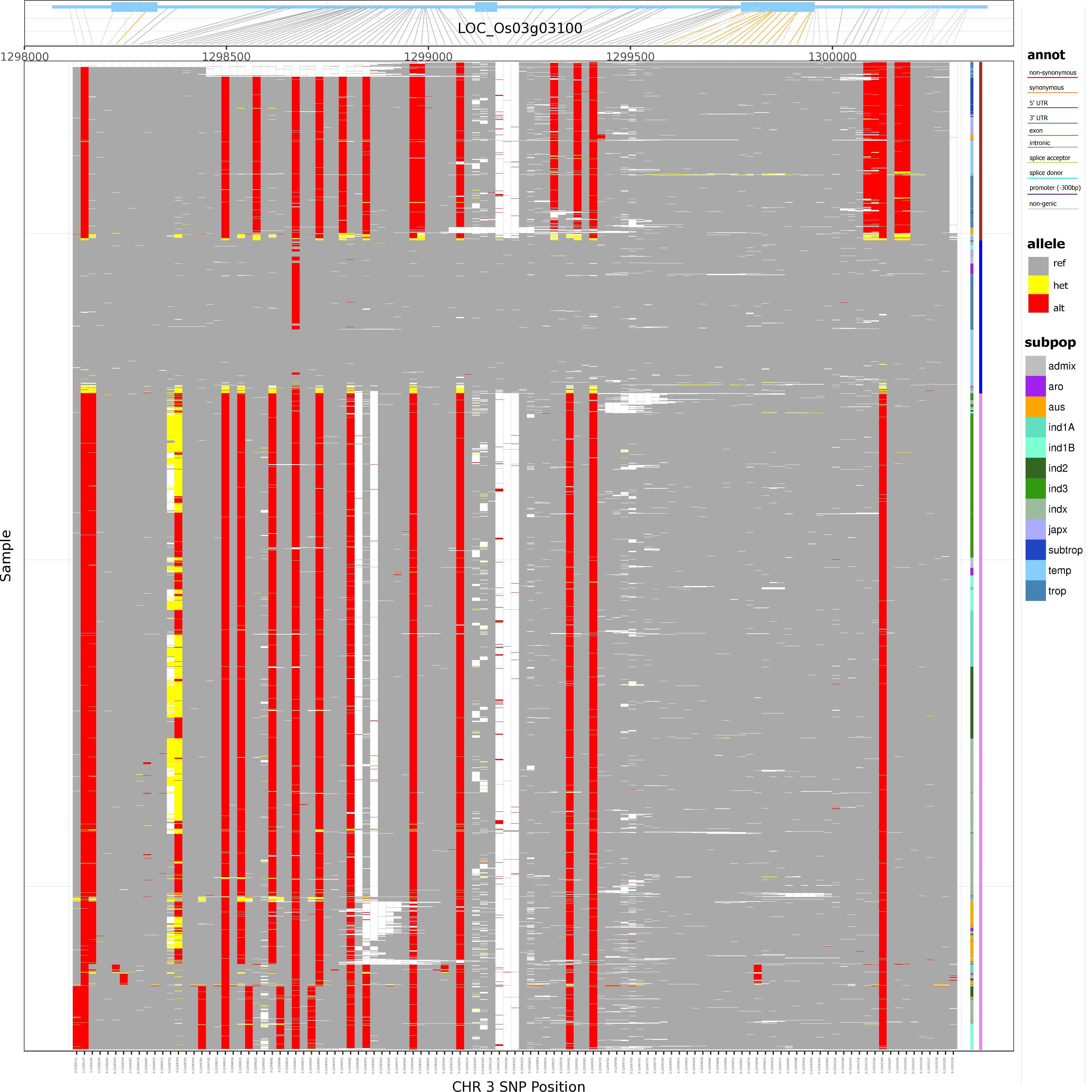

Supplement: Supplementary Figure 4 — Haplotype analysis using SNPs identified in LOC_Os03g03100 (OsMADS50) using 3,024 sequenced genomes from the 3K Rice Genotyping Project. [file Image_4.JPEG]
