# Supplementary material for: Genomic Regions and Floral Traits Contributing to Low Temperature Tolerance at Young Microspore Stage in a Rice (Oryza sativa L.) Recombinant Inbred Line Population of Sherpa/IRAT109
Source: Front Plant Sci. 2022 Apr 29;13:873677. doi: 10.3389/fpls.2022.873677 (PMC9100824; doi:10.3389/fpls.2022.873677)
Supplement: Supplementary Table 1 — Summary statistics for whole-genome sequence analysis of IRAT109 and Sherpa using Illumina NovaSeq 6000. [file Data_Sheet_1.docx]

Table S1. Summary statistics for whole genome sequence analysis of IRAT109 and Sherpa using Illumina NovaSeq 6000

| **Criteria** | **IRAT109** | **Sherpa** |
| --- | --- | --- |
| Length (bp) | 2 x 90 | 2 x 150 |
| QC Passed Reads | 109,016,734 | 69,192,646 |
| Duplicate Reads | 7,562,638 | 4,401,736 |
| Mapped Reads | 104,471,105 | 67,951,544 |
| Properly Paired | 99,387,811 | 54,326,698 |
| Duplicates (%) | 6.9 | 6.4 |
| Mapped (%) | 95.8 | 98.2 |
| Properly Paired (%) | 91.2 | 78.5 |
| Depth of Coverage | 22.1 | 21.1 |

Table S2. Variants identfied using IRAT109 and Sherpa resequencing data per chromosome

| **Chromosome** | **Length** | **Variants** | **Variant Rate** |
| --- | --- | --- | --- |
| 1 | 43,270,923 | 132,376 | 326 |
| 2 | 35,937,250 | 73,468 | 489 |
| 3 | 36,413,819 | 60,428 | 602 |
| 4 | 35,502,694 | 111,315 | 318 |
| 5 | 29,958,434 | 84,705 | 353 |
| 6 | 31,248,787 | 105,105 | 297 |
| 7 | 29,697,621 | 97,457 | 304 |
| 8 | 28,443,022 | 94,148 | 302 |
| 9 | 23,012,720 | 45,610 | 504 |
| 10 | 23,207,287 | 107,658 | 215 |
| 11 | 29,021,106 | 87,427 | 331 |
| 12 | 27,531,856 | 91,848 | 299 |
| Sy | 592,136 | 1,454 | 407 |
| Un | 633,585 | 1,122 | 564 |
| Total | 374,471,240 | 1,094,121 | 342^a^ |

^a^-Mean variant rate across chromosomes

Table S3. Variants identified in LOC_Os03g03100 (*OsMADS50*) gene between Sherpa and IRAT109

| SITE NAME | CHR | POS | VARIANT | IRAT109 | SHERPA | TYPE | ANNOTATION |
| --- | --- | --- | --- | --- | --- | --- | --- |
| S3_1298618 | 3 | 1298618 | CTA/CAATA | CAATA | CTA | ins | Intron variant |
| S3_1298956 | 3 | 1298956 | G/A | A | G | snp | Intron variant |
| S3_1299022 | 3 | 1299022 | G/A | A | G | snp | Intron variant |
| S3_1299068 | 3 | 1299068 | CA/CTC | CTC | CA | complex | Intron variant |
| S3_1299527 | 3 | 1299527 | G/A | A | G | snp | Intron variant |
| S3_1299562 | 3 | 1299562 | G/A | A | G | snp | Intron variant |
| S3_1300188 | 3 | 1300188 | A/C | C | A | snp | 5 prime UTR/premature start codon gain variant |
| S3_1300479 | 3 | 1300479 | C/G | G | C | snp | Upstream gene variant |
| S3_1300994 | 3 | 1300994 | AC/TT | TT | AC | complex | Upstream gene variant |
| S3_1301325 | 3 | 1301325 | C/A | A | C | snp | Upstream gene variant |
| S3_1301517 | 3 | 1301517 | C/A | A | C | snp | Upstream gene variant |
| S3_1301571 | 3 | 1301571 | C/T | T | C | snp | Upstream gene variant |
| S3_1301605 | 3 | 1301605 | T/G | G | T | snp | Upstream gene variant |
| S3_1301804 | 3 | 1301804 | G/A | A | G | snp | Upstream gene variant |
| S3_1301812 | 3 | 1301812 | C/T | T | C | snp | Upstream gene variant |
| S3_1301870 | 3 | 1301870 | C/T | T | C | snp | Upstream gene variant |
| S3_1301922 | 3 | 1301922 | G/A | A | G | snp | Upstream gene variant |

CHR- chromosome; POS- position in bp; ins- insertion; snp- single nucleotide polymorphism

Table S4. Heirarchy, RAPDB locus ID, and description of co-expressed genes with *OsMADS50*.

| **Hierarchy** | **LocusID** | **Description** |
| --- | --- | --- |
| 0 | Os03g0122600 | Transcription factor, MADS-box domain containing protein. |
| 1 | Os05g0495600 | Similar to Calcium-transporting ATPase 4, plasma membrane-type (EC 3.6.3.8) (Ca(2+)-ATPase isoform 4). |
| 1 | Os06g0217300 | Similar to Transcription factor MADS55. |
| 1 | Os06g0228500 | Amino acid/polyamine transporter II family protein. |
| 2 | Os01g0686200 | UDP-glucuronosyl/UDP-glucosyltransferase family protein. |
| 2 | Os04g0509500 | Similar to Ammonium transporter Amt1;1 (Fragment). |
| 2 | Os05g0586200 | GH3 auxin-responsive promoter family protein. |
| 2 | Os06g0225300 | Similar to SERK1 (Fragment). |
| 3 | Os01g0752100 | Cyclin-like F-box domain containing protein. |
| 3 | Os01g0842200 | Similar to Scarecrow-like 9 (Fragment). |
| 3 | Os01g0866400 | Similar to Fructose-1,6-bisphosphatase (EC 3.1.3.11) (Fragment). |
| 3 | Os01g0940000 | FAD linked oxidase, N-terminal domain containing protein. |
| 3 | Os02g0468400 | Lipid-binding START domain containing protein. |
| 3 | Os04g0509600 | Similar to Ammonium transporter Amt1;2 (Fragment). |
| 3 | Os06g0691400 | Similar to IAA-amino acid conjugate hydrolase-like protein (Fragment). |
| 3 | Os09g0553900 | Conserved hypothetical protein. |
| 3 | Os11g0191400 | ATP-NAD/AcoX kinase family protein. |
